# Revealing the diversity of extracellular vesicles using high-dimensional flow cytometry analyses

**DOI:** 10.1038/srep35928

**Published:** 2016-10-27

**Authors:** Geneviève Marcoux, Anne-Claire Duchez, Nathalie Cloutier, Patrick Provost, Peter A. Nigrovic, Eric Boilard

**Affiliations:** 1 Centre de Recherche du Centre Hospitalier Universitaire de Québec, Faculté de Médecine de l’Université Laval, Département de microbiologie et immunologie, Québec, QC, Canada; 2 Department of Medicine, Division of Rheumatology Immunology and Allergy, Brigham and Women’s Hospital and Harvard Medical School, Boston, MA, USA

## Abstract

Extracellular vesicles (EV) are small membrane vesicles produced by cells upon activation and apoptosis. EVs are heterogeneous according to their origin, mode of release, membrane composition, organelle and biochemical content, and other factors. Whereas it is apparent that EVs are implicated in intercellular communication, they can also be used as biomarkers. Continuous improvements in pre-analytical parameters and flow cytometry permit more efficient assessment of EVs; however, methods to more objectively distinguish EVs from cells and background, and to interpret multiple single-EV parameters are lacking. We used spanning-tree progression analysis of density-normalized events (SPADE) as a computational approach for the organization of EV subpopulations released by platelets and erythrocytes. SPADE distinguished EVs, and logically organized EVs detected by high-sensitivity flow cytofluorometry based on size estimation, granularity, mitochondrial content, and phosphatidylserine and protein receptor surface expression. Plasma EVs were organized by hierarchy, permitting appreciation of their heterogeneity. Furthermore, SPADE was used to analyze EVs present in the synovial fluid of patients with inflammatory arthritis. Its algorithm efficiently revealed subtypes of arthritic patients based on EV heterogeneity patterns. Our study reveals that computational algorithms are useful for the analysis of high-dimensional single EV data, thereby facilitating comprehension of EV functions and biomarker development.

Extracellular vesicles (EV) are small membrane vesicles released by cells into the extracellular milieu. They are subdivided into three major groups, primarily based on the process underlying their release. Exosomes are produced by exocytosis of multivesicular bodies and have a diameter that ranges between approximately 50–150 nm. Microvesicles (MV), also termed microparticles or ectosomes, are vesicles of approximately 100–1000 nm in diameter generated by cytoplasmic membrane budding and fission. Apoptotic bodies are released by apoptotic cells and generally possess dimensions larger than 1000 nm^1^,^2^,^3^. However, it should be noted that these definitions are still subject to change, as larger exosomes (up to 250 nm) have been described, and apoptotic cells also release exosome-like vesicles^3^,^4^. Hence, the term EV is increasingly utilized, as it more liberally encompasses all vesicle types released by cells.

As EVs carrying components from donor cells can be internalized by cellular recipients, they are implicated in intercellular communication^5^,^6^,^7^. Eukaryotic, and prokaryotic cells produce EVs, suggesting that this mechanism is well-conserved throughout evolution, which points to its significance. Furthermore, EVs are induced by cell activation, and EV levels in biological fluids are altered in different pathologies, such as cancer, rheumatic diseases, neurodegenerative disorders, organ damage, and infectious diseases^2^,^3^,^8^. Hence, EVs appear to be potent biomarkers, and their detection in blood and other biological fluids is improving with the recent establishment of pre-analytical conditions, standardization of quantification approaches, and development of more efficient means of detection^9^,^10^,^11^,^12^,^13^,^14^,^15^.

EVs (more specifically MVs) have been described in the blood of healthy individuals. They mainly comprise MVs derived from platelets and red blood cells (RBC)^16^,^17^,^18^,^19^. Cell surface markers allow the cellular origin of MVs to be distinguished as CD41a and CD235a are detected on the surface of platelets and RBC MVs, respectively. Studies show that megakaryocytes (MK) can also generate CD41a^+^-MVs, and that MVs in blood derived from MKs can be distinguished from those of platelets by surface expression of P-selectin (CD62P), lysosomal-associated membrane protein 1 (LAMP-I) and immunoreceptor-based activation motif (ITAM) receptors^16^,^20^,^21^. Thus, cell lineage markers provide useful information for determining the cellular source of EVs.

EV heterogeneity is further complicated by the presence or absence of other components. Phosphatidylserine (PS) exposure by EVs, which permits identification of the latter with probes conjugated to PS-binding proteins such as annexin V and lactadherin, can in fact appear undetectable on a substantial proportion of EVs^16^,^22^,^23^,^24^. EV subpopulations may contain active proteasome, organelles (*e.g*. mitochondria), and may undergo post-translational modification in disease, such as citrullination in inflammatory arthritis^4^,^16^,^23^,^25^,^26^,^27^. Furthermore, EVs are decorated with autoantibodies in autoimmune diseases such as systemic lupus erythematosus (SLE) and rheumatoid arthritis (RA)^16^,^23^,^28^, and different platelet stimuli were shown to trigger release of different EV subpopulations^18^,^24^. In summary, EVs are highly heterogeneous, and the recognition of EV subtypes is necessary for the comprehension of EV functions and consistent design of biomarkers.

Seminal research has established optimal pre-analytical conditions for the preparation of samples for EV analyses^12^,^29^,^30^. Flow cytometry (FCM) remains the most convenient methodology for assessment of EVs in biological fluids, especially when a large sample number is analyzed, and when further characterization is required using multiple markers on hundreds of thousands of EVs simultaneously. Whereas traditional flow cytometers were not designed for the detection of such small vesicles, the recent development of high sensitivity FCM (hs-FCM) has greatly improved investigators’ capabilities of analyzing EVs and developing biomarkers based on EV heterogeneity^10^,^12^,^13^,^15^,^23^. However, even with up-to-date approaches for the detection of EVs, investigators are left with tremendous amounts of high-dimensional datasets that are impossible to interpret using traditional gating on bivariate dot plots. Furthermore, with more powerful methods of detection, distinguishing potentially relevant, but rare, subpopulations of EVs from the generally important non-specific background is a recognized issue^12^. There is currently no study that reports a means of facilitating interpretation of flow cytofluorometric analyses of EVs. Thus, with the rapidly growing improvements in detection technologies, and recognition of the vast heterogeneity of EVs, the development of methods to objectively interpret multiple single-EV parameters is urgent.

Scientists implicated in the study of EVs are, of course, not the first to face this sort of challenge. For instance, in immunology, an understanding of the functions of discrete immune cells has evolved greatly since the discovery of the different immune cell subtypes. It is now well-accepted that T lymphocytes are heterogeneous and plastic, with CD4 and CD8 expression, and the identification of several subtypes such as Th1, Th2, Th17, regulatory T cells and follicular-helper T cells^31^. Moreover, dendritic cells^32^,^33^, monocytes, macrophages^34^ and neutrophils^35^,^36^ are subdivided into different classes based on surface receptor expression and density. From this perspective, traditional methods to analyze multidimensional single-cell data are inadequate, as they are subjective and restricted to our actual knowledge of the cellular phenotypes.

Spanning-tree progression analysis of density-normalized events (SPADE) is a versatile computational approach that can be applied to mass and FCM data^37^. SPADE does not require manual gating, which is considered more subjective^37^,^38^, and allows the visualization of rare cell types, which would be lost or simply excluded if they were unexpected. Also, it provides a global overview of cellular heterogeneity by showing a continuity of phenotype, instead of clustering events in an independent and strictly-defined population. Those features were successfully used to characterize cell populations in different contexts^37^,^39^,^40^,^41^,^42^,^43^.

In this present study, we successfully used SPADE algorithms to build an EV hierarchy for the appreciation of EV diversity in biological fluids.

## Materials and Methods

### Ethics

The study was approved by the medical ethics committee of CHU de Québec and Université Laval. Methods were carried out in accordance with the approved guidelines.

Platelets and RBC were obtained from the citrated blood of healthy human volunteers under informed consent according to a protocol approved by an Institutional Review Board (Centre de Recherche du Centre Hospitalier Universitaire de Québec). Synovial fluid (SF) was obtained from volunteers with rheumatoid arthritis (RA) with approval of the ethic committee (Brigham and Women’s Hospital) and under informed consent.

### Platelets

Platelets were isolated after centrifugation of blood (282× *g* for 10 min) at room temperature (RT). Platelet-rich plasma (PRP) was obtained after an additional centrifugation of the supernatant at 600× *g* for 3 min at RT. The supernatant was then centrifuged at 1,300× *g* for 5 min at RT and the platelet-containing pellet was resuspended in Tyrode’s buffer (pH 7.4). Platelets were counted (Cellometer AutoM10; Nexcelom Bioscience) and adjusted to a density of 100 × 10^6^ cells/ml in Tyrode’s buffer (pH 7.4). For platelet EVs, platelets were stimulated with thrombin (0.5 U/ml, Sigma) for 1 h at 37 °C after addition of 5 mM of calcium. Platelet activation was stopped by addition of 20 mM of EDTA, and remnant platelets were removed by centrifugation at 1,300× *g* for 5 min at RT, performed twice.

### Platelet-free plasma (PFP)

PRP from healthy volunteers was prepared as above without any stimulation and was centrifuged at 2,500× *g* for 20 min at RT. Then, the supernatant was centrifuged twice at 3,200× *g* for 5 min when platelet-free plasma (PFP) was required.

### Red blood cells (RBC)

Red blood cells were isolated after centrifugation of blood (282× *g* for 10 min at RT). Platelet-rich plasma and buffy coat fractions were eliminated to conserve the RBC pellet. Red blood cells were counted (Cellometer AutoM10), adjusted to a density of 100 × 10^6^ cells/mL in Tyrode’s buffer (pH 7.4) and contained less than 0.004% (n = 3) contaminating platelets. For RBC EVs, 200 μl of this preparation was added to 50 ml of distilled water (filtered through a 0.22-μm membrane) for 5 min, and 5 ml of PBS 10X (filtered through a 0.22-μm membrane) was then added to stop the hypotonic reaction. Remnant RBC were removed by centrifugation at 1,300× *g* for 5 min at RT^13^.

### Characterization of EVs

#### Hs-FCM approach

All the analyses were performed on a BD Canto II Special Order Research Product (BD Biosciences) equipped with a small particle option, as described previously^13^,^23^. The forward scatter (FSC) on this dedicated equipment is coupled to a photomultiplier tube (PMT) with a 488 nm solid state, 100 mW output blue laser (rather than the conventional 20 mW), and includes a 633 nm HeNe, 20 mW output red laser and a 405 nm solid state diode, 50 mW output violet laser. The hs-FCM includes an FSC-PMT and a Fourier optical transformation unit, which reduces the background/noise and increases the angle of diffusion, thereby enhancing the detection of small-diameter particles.

FCM performance tracking was performed daily before all analyses using the BD cytometer setup and tracking beads (BD Biosciences, San Jose, CA, USA). The assigned voltage for FSC-PMT was 300 volts (V). For side scatter (SSC), the assigned voltage was 460 V and the threshold was 200. Voltage was set to 360 V for FITC, 450 V for PE-Cy7, 500 V for Deep Red (or APC), 500 V for PE, 500 V for Alexa Fluor 700 (used only for the counting beads) and 305 V for V450. Acquisition was performed at low speed (~10 μl/min) and, to remain quantitative, a known quantity of (fluorescent) polystyrene microsphere (15-μm diameter: Polysciences, PA, USA) was added to each tube, and a constant number of beads detected on the basis of (auto) fluorescence was acquired for each sample throughout all the study. Silica particles (Kisker Biotech GmbH & Co. Steinfurt, Germany) of known dimensions (100 nm, 500 nm and 1 μm in diameter) were used for instrument set-up standardization^13^,^23^. Fluorophores implicating distinct lasers were intentionally chosen to minimise compensation. For every performed experiment, the only necessary compensation was 5.00% for APC-V450.

#### Detection of platelets, RBC and EVs by flow cytometry

Platelets and EVs were prepared as above and 5 μL per sample was labeled in a total reaction volume of 100 μL (as indicated in Table S1) at 37 °C for 30 min. Then, the sample was diluted by adding 400 μl of the labeling buffer prior analysis by hs-FCM. RBC-derived samples were labeled as described above with the exception of MitoTracker, which was omitted given the known absence of mitochondria in RBC. For Triton or EDTA treatment, samples (5 μL) were incubated 30 min at RT with 0.05% Triton X-100 or 50 μM EDTA (with PBS instead of Annexin V buffer for EDTA) before labeling. For the ultracentrifugation treatment, samples were centrifuge at 100 000 g × 1 h at 20 °C to pellet EVs, and the supernatant was labeled as presented above.

#### Plasma EV diversity with overlapping staining panels

EVs contained in platelet-free plasma (5 μL for 100 μL reaction) were labeled as indicated in Table S1 with the four different cocktails (5a, b, c and d). The reaction was stopped by adding 400 μl of the labeling buffer.

#### EV diversity in RA synovial fluid

Freshly obtained SF, collected without anticoagulant, was centrifuged at 1,900 × *g* for 30 min at 4 °C to remove leukocytes and then stored at −80 °C. EVs present within the SF (5 μL for 100 μL reaction) were labeled as indicated in Table S1 and the reaction was stopped by adding 400 μl of the labelling buffer.

#### Immunoblotting

Cells were pelleted at 1,300× g for 5 min: EVs derived from platelets and RBC and those present in plasma were centrifuged at 18,000× g for 90 min^5^ and processed in lysis buffer (20 mM Tris HCl pH 7.8, 1.25 mM EDTA, 0.5% Triton X-100, 0.5% NP-40, 120 mM NaCl, 2 mM phenylmethylsulfonyl fluoride.

Lysate protein content was measured using a bicinchoninic acid protein assay kit (Fisher). Proteins (10 μg) were separated by 10% SDS-PAGE, transferred to a polyvinylidene difluoride membrane and the candidate proteins were detected using antibodies against platelet MV marker CD41a (Abcam used at 1/1,000), an enzyme reportedly present in platelet MVs; 12-lipoxygenase (12LO) (Santa Cruz, used at 1 μg/ml); cytoskeleton marker β-actin (Sigma, clone AC-15 used at 1/15,000); the EV markers, tumor susceptibility 101 (TSG101)(Abcam, clone 4A10 used at 0.1 mg/ml) and ALG-interacting protein X (ALIX) (Santa Cruz, clone 3A9 used at 1 μg/ml); the RBC marker CD235a (Santa Cruz, clone YTH89.1 used at 1 μg/ml); and the mitochondrial markers, voltage-dependent anion channel (VDAC) (Cell Signaling, used at 11.6 μg/ml) and translocase of outer mitochondrial membrane TOMM-22 (Abcam, used at 2.5 μg/ml)^5^,^44^. The PVDF membranes were incubated with peroxidase-conjugated antibodies recognizing the primary antibodies (Jackson ImmunoResearch, used at 0.08 μg/ml).

#### Size analysis

Silica beads were diluted 1/100 in PBS 1X (filtered through a 0.22-μm membrane) and analyzed by Zetasizer Nano S (Malvern Instruments, Ltd., Malvern, UK).

### Statistical analyses

The results are presented as mean ± SEM, and were analyzed with Prism 6 (GraphPad Software, CA, USA).

### SPADE analyses

The pre-compiled standalone version of SPADE-3 for Mac without Matlab was downloaded at http://pengqiu.gatech.edu/software/SPADE/. FCS files were exported from BD FACSDiva™ software (BD Bioscience) in FCS 3.0 format and analyzed using FlowJo (FlowJo, LLC, OR, USA) software to exclude counting beads and events with dimensions smaller than 100-nm silica beads. Specific details for each SPADE analysis are provided within the results section.

## Results

### Optimization and validation of high sensitivity flow cytofluorometric methods for the detection of EVs

In the first set of experiments, we validated that platelets, RBC, and their daughter EVs, were efficiently resolved by hs-FCM. EVs were not pelleted prior to hs-FCM analyses, given the reported deleterious impact of this procedure on EV integrity^45^. Furthermore, as one goal of this study was to appreciate EV diversity, we chose to maintain the complexity of our EV preparations by avoiding exosome and MV enrichment. Therefore, EV preparations comprised a mixture of exosomes and MVs derived from platelets and RBC, and the hs-FCM conditions were optimally designed to detect EVs larger than 100 nm silica beads.

Given that size is a factor of interest in these analyses, we used microspheres of known dimensions to standardize the instrument setup. Whereas polystyrene microspheres are frequently utilized for the determination of size, their refraction index (1.59) differs considerably from that of membrane vesicles (1.39)^13^,^46^,^47^. Hence, while size, shape, surface roughness, granularity and the angle of collection impact light scattering, the intensity of the scattered light greatly depends on the refraction index for particles with dimensions smaller than the wavelength of light (in this case 488 nm). Thus, for our analyses, we used silica beads, which have a refraction index (1.42)^13^,^46^,^47^ closer to that of membrane vesicles, to establish the lower limit of the EV gate, and we included intact platelets and RBC to ensure that they were efficiently distinguished from their respective daughter EVs.

**Figure 1:**
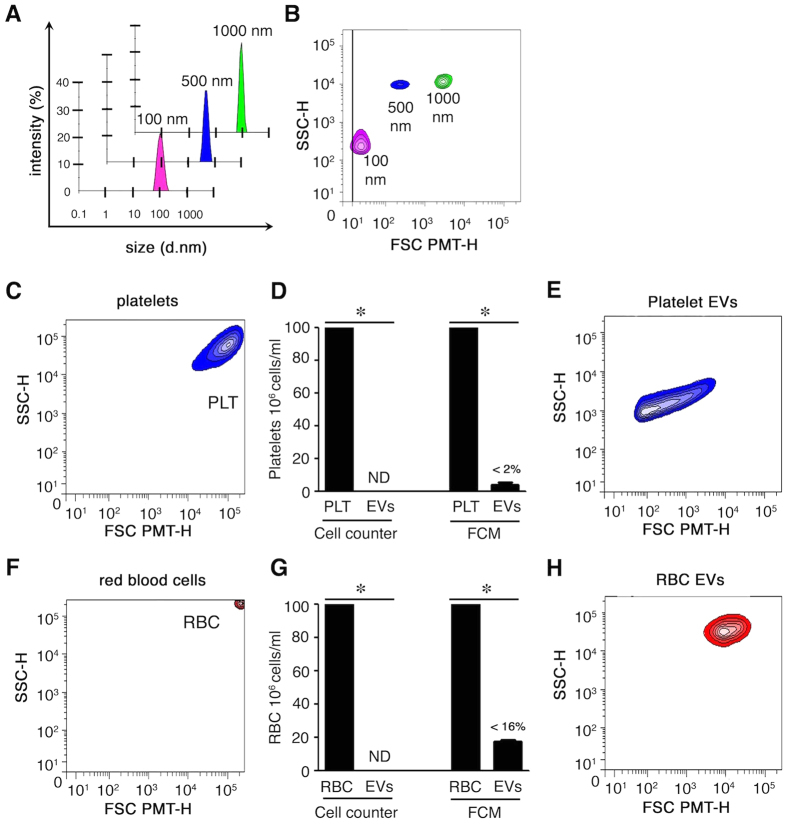
Characterization of extracellular vesicles. (**A**) Size evaluation of silica beads by nanosizer based on dynamic light scattering. (**B**) Design of a lower EV gate by hs-FCM based on acquisition of silica beads of various sizes (100 to 1000 nm). (**C**) Portrayal of non-activated platelets (PLT). (**D**) Remnant platelets detected in the EV fraction (after stimulation) using a cell counter and FCM (<2%). ND: not detected. (**E**) EVs from preparations cleared of any remnant platelets by centrifugations were analyzed by hs-FCM. Representative of 3 different donors. (**F**) Portrayal of RBC analyzed by FCM. (**G**) Remnant RBCs detected in the EV fraction (after stimulation) using cell counter and FCM (<16%). ND: not detected. (**H**) EVs from preparations cleared of any remnant RBCs by centrifugations were analyzed by hs-FCM. Representative of 3 different donors, *P < 0.05.

We validated that silica microspheres ranging from 100–1000 nm in diameter (Fig. 1A) were efficiently resolved by hs-FCM (Fig. 1B). Resting platelets were as expected larger than 1000 nm silica beads (Fig. 1C)^48^. To generate platelet EVs, platelets were triggered by thrombin and remnant platelets were removed by centrifugation. In these preparations, platelets were undetectable (Fig. 1D), and platelet EVs were detected by hs-FCM (Fig. 1E), and were clearly distinguishable from intact platelets (compared to Fig. 1C). As expected, RBC (mean diameter between 7–8 μm) appeared much larger than platelets in our hs-FCM analyses (Fig. 1F), and were triggered to release EVs by osmotic shock. RBCs were largely absent from EV preparations (Fig. 1H). RBC EVs were efficiently distinguished from intact RBC (Fig. 1H), although they displayed apparent larger dimensions than platelet EVs in hs-FCM (compared to Fig. 1E). This observation will not be investigated further in the present study. Thus, small EVs are detected in our hs-FCM analyses.

Fluorochrome-conjugated probes and antibodies can form submicron aggregates in solution, which can be mistakenly interpreted as EVs by hs-FCM^49^. Furthermore, multiple EVs can be detected simultaneously if present at a too elevated concentration or analyzed at high acquisition speed, a process called coincidence or swarm that compromises the interpretation of EV multicolor labeling^47^. To ensure that genuine EVs were detected, and that no signals arose from aggregated fluorochromes, we used an established detergent assay^49^,^50^. Under these conditions, the membrane moiety of the EVs is dissolved by Triton X-100 treatment while protein aggregates are left intact^13^,^23^,^49^,^50^. In addition, the specificity of PS recognition by annexin V-conjugated fluorochromes, which is a calcium-dependent event, was confirmed by calcium chelation using EDTA^13^,^22^. As EVs can be pelleted by centrifugation, we also verified that no EVs were detected in fluids after ultracentrifugation.

**Figure 2:**
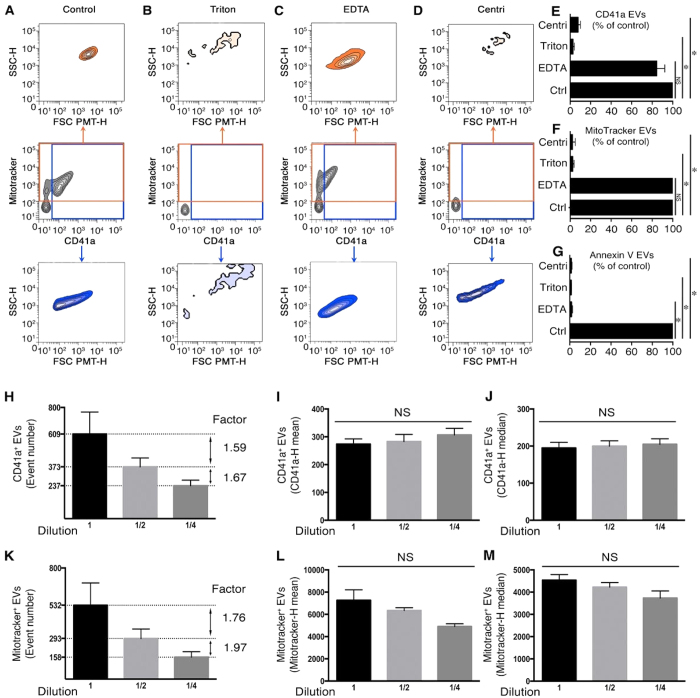
Detection of platelet EV subpopulations by hs-FCM. (**A**) Representative SSC-H (granularity) and FSC-PMT-H (relative size) dot plots of platelet EVs detected using MitoTracker and fluorochrome-conjugated antibodies directed against CD41a in the absence of treatment (control). (**B**–**D**) FSC-PMT/SSC portrayals of platelet EVs detected with MitoTracker and antibodies directed against CD41a after treatment with 0.05% Triton X-100 (**B**) 50 μM EDTA (**C**) or after clearance of EVs using ultracentrifugation (centri) (**D**). Total MitoTracker^+^ EVs are presented in the orange gate (middle panel), and their relative size and granularity is displayed in the upper panel. Total CD41a^+^ EVs are presented in the blue gate (middle panel), and their relative size and granularity is displayed in the lower panel. Data are representative of 3 independent experiments. (**E**,**F**) Sensitivity of CD41a (**E**) MitoTracker (**F**) and annexin V (**G**) EVs to clearance by ultracentrifugation (centri), Triton and EDTA, presented as % of untreated (Ctrl). (**H**–**M**) CD41a^+^ and MitoTracker^+^ EVs were serially diluted twice (2-fold dilution) and quantitatively analyzed by hs-FCM using counting microspheres. Their concentration (**H**,**K**), the mean of fluorescence (**I**,**L**) and the median of fluorescence (**J**,**M**), are presented. Data are presented as mean ± SEM of 3 independent experiments, *P < 0.05 compared with the control (Ctrl); NS: Non significant; Wilcoxon test.

Platelet EVs may contain mitochondria, and can express surface CD41a and PS^5^,^25^. Thus, platelet EVs, detected by a combination of mitochondrial dye MitoTracker, anti-CD41a antibody and annexin V (Fig. 2A), were treated with detergent (Fig. 2B), or EDTA (Fig. 2C). Furthermore, all the fluorescent probes were incubated with fluids that underwent ultracentrifugation (Fig. 2D). Under these conditions, the vast majority of EVs positive for CD41a (Fig. 2B,D,E), MitoTracker (Fig. 2B,D,F), and annexin V (Fig. 2G), were eliminated by detergent and centrifugation. Conversely, EDTA primarily affected annexin V labeling (Fig. 2G), with only a modest impact on CD41a (Fig. 2C,E) and MitoTracker signals (Fig. 2C,F), thereby validating the specificity of our multicolor labeling of platelet EVs and further confirming the heterogeneity of EVs.

We next verified the absence of coincidence in our hs-FCM conditions and validated our quantitative strategies. In the absence of coincidence, the concentration of EVs should be reduced according to dilution factors, while the mean fluorescence intensity should remain constant^13^. We confirmed the lack of coincidence, as the concentrations of EVs positive for CD41a (Fig. 2H), MitoTracker (Fig. 2K) and annexin V (data not shown for platelet EVs) were consistently reduced without any impact on the mean and median fluorescence intensity (Fig. 2I,J,L,M). Using anti-CD235a and annexin V-conjugated probes, we also confirmed the specificity of our signals and the absence of coincidence in our flow cytofluorometric acquisitions of RBC EVs (Figure S1).

Depending on their mechanism of release, EVs may contain distinct sets of proteins and organelles^44^. Of note is that immunoblotting confirmed that our EV preparations contained proteins reportedly present in EVs^5^,^25^,^44^. As expected, CD235a was absent in EVs derived from platelets, whereas the surface protein CD41a, the cytosolic platelet 12-LO, the cytoskeleton protein actin, the EV proteins TSG101 and ALIX, and the mitochondrial proteins VDAC and TOMM-22 were detected in platelet EVs. CD41a, platelet 12-LO, and mitochondrial markers were absent in RBC EVs, whereas cytoskeleton and EV markers were detected (Figure S2). Together, these observations confirm that our strategies are optimal for the establishment of optimal high-dimensional dataset analyses of EVs.

### Analysis of RBCs, platelets and their EVs using SPADE

Contrary to traditional gating analysis, where gates must be manually designed, SPADE uses topological methods to reveal distinct populations of cells from high-dimensional data sets^37^,^38^,^43^, and also equally represents rare and abundant cell types (and potentially EVs). This is important, because rare, but biologically relevant EVs, might be masked if outnumbered by background or noise; a particularly frequent issue in FCM analyses of EVs. Events (*e.g*. cells, or potentially EVs here) that share similitudes on the basis of marker expression are clustered within the same node. Each node can be colored according to their median intensity for a given marker expression (low to high; blue to red, respectively) and the size of the node reflects the number of events that it contains^43^. Nodes that belong to the same branch on the tree are more likely to be related to each other than nodes found on different branches, and the length of the branches is determined automatically by the program^43^. Thus, using multiple fluorescent markers, in addition to light scatter (FSC-PMT and SSC), it might become possible to identify groups of EVs that are similar with respect to each measured parameter.

RBCs, platelets, and their respective EVs generated *in vitro*, as above (n = 3 blood donors), were detected by hs-FCM on the basis of expression of CD41a, CD235a, PS exposure, mitochondrial content, size (FSC-PMT) and inner complexity (SSC). FCS files were pre-analyzed to exclude counting beads (Figure S3A) and events smaller than 100 nm silica beads (Figure S3B and Fig. 1B). The files were used to build the SPADE tree with the following markers: FSC-H (for cells), FSC-PMT-H, SSC-H, MitoTracker-H, CD41a-H, CD235a-H and annexin V-H. An inverse hyperbolic sine transformation with cofactor 150 was applied in order to scale the data, and the maximum allowable cells/EVs in the pooled down-sampled data was set to 50,000. The outlier was set to the 1^st^ percentile of local densities and the target density was set such that a fixed number of 20,000 cells would remain. The number of desired clusters was 200, as a high EV heterogeneity was expected, and the K-means algorithm was chosen as the clustering parameter.

**Figure 3:**
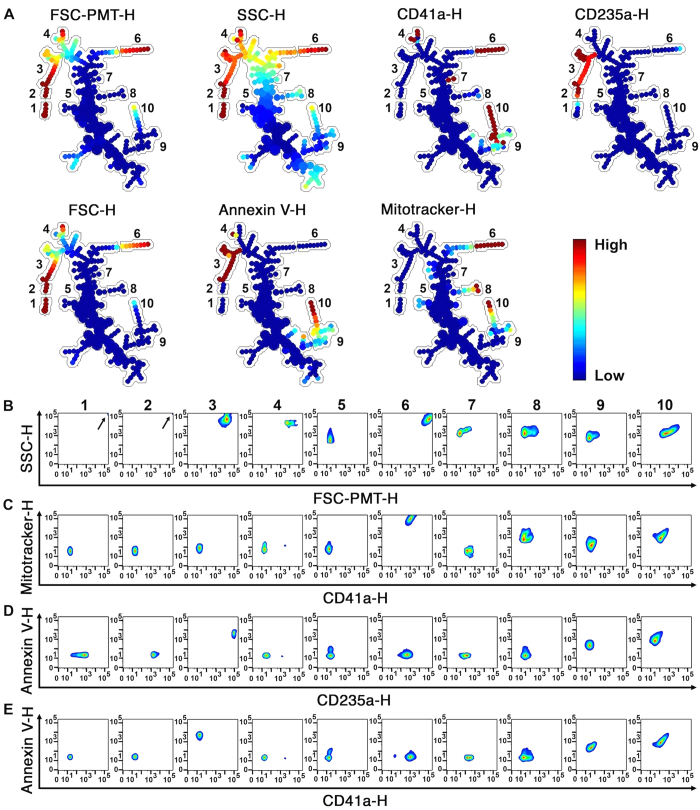
SPADE analysis of human blood cells and EVs. (**A**) SPADE tree derived from RBC, platelets and EVs from healthy human blood cells using FCS files obtained by hs-FCM analyses. Each tree is colored according to the indicated marker. Mean fluorescence intensity ranging from low (blue) to high (red) was used to identify 10 different subpopulations (1*–*10) described in the text. (**B**–**E**) Events from the different subpopulations identified in trees were represented by classical analysis with bivariate plots showing (**B**) size and granularity, (**C**) CD41a and MitoTracker expression, (**D**) annexin V and CD235a expression or (**E**) CD41a and annexin V expression. (**B**) Arrow indicates highest cellular population in FSC-PMT-H and SSC-H. Data were generated using cells and EVs from 3 independent experiments. FCS data from the independent experiments were integrated by SPADE to generate trees.

Using the semi-automated annotation tool (button “Auto Suggest Annotation”), which relies on all markers used to build the tree, a tree was automatically generated (Fig. 3A), distinguishing 10 sub-populations (namely 1*–*10). The first autosuggestion revealed a strong difference in CD235a expression, size and inner complexity, and isolated the CD235a (RBC) high branch (1–3) from the rest of the tree. A second autosuggestion highlighted a subpopulation (6) presenting high expression of CD41a, MitoTracker, SSC, FSC and FSC-PMT, which correspond to platelets. The three subsequent autosuggestions revealed PS-expressing EVs produced from platelets (9–10) and from RBC (3), which were also smaller than their mother cells. As (1*–*2) subpopulations showed a homogeneous distribution for every marker except for CD235a expression, the software suggested division of this branch into two. Autosuggestions also distinguished mitochondria-containing EVs that did not present RBC (CD235a^−^) or platelet (CD41a^−^) markers (subpopulation 8), and allowed the partition of subpopulations (9–10) according to CD41a, MitoTracker and PS expression. It is important to note that these populations were objectively identified without any gating or prior knowledge. Only subpopulations (4) and (7) were drawn manually, mainly because of their bright intensities in distinctive markers.

The (1–10) subpopulations were then annotated, and the SPADE tree was interpreted. Subpopulation (1) includes cells (high intensity for FSC-PMT-H, FSC-H and SSC-H) that were not RBCs (low expression of CD235a) or platelets (low expression of CD41a), potentially representing a low number of contaminating leukocytes or RBC ghosts generated by RBC activation. Subpopulation (2) contains RBCs, which show high intensity for FSC-PMT, FSC and SSC and also high expression of CD235a markers, but low expression of CD41a. Subpopulation (3) contains RBC EVs with intermediate intensity for FSC-PMT, FSC and SSC, high expression of CD235a and low expression of CD41a. All RBC EVs detected in those samples exposed PS (high intensity for annexin V expression). With a low expression of all 7 markers, the (5) subpopulation was annotated as background, although it might also contain EVs left unidentified using this set of markers. Subpopulation (6) contains platelets, which show relatively high light scatter for FSC-PMT, FSC and SSC and expression of CD41a and MitoTracker. Subpopulation (8) showed low expression of all markers except for MitoTracker, suggesting that they might be naked mitochondria or mitochondria encapsulated in EVs lacking expression of CD41a. Subpopulations (7, 9 and 10) represent platelet EVs (intermediate CD41a expression and SSC, low CD235a expression and low light scatter for FSC-PMT and FSC). Subpopulation (4) includes EVs with variable expression levels of CD41a (low to high), with those presenting the brightest CD41a intensity representing 1,15+/-1,99% of this subpopulation (data not shown). Subpopulation (10) includes EVs containing mitochondria (high MitoTracker expression), with variable exposure of PS (intermediate to high intensity annexin V binding). More than 40 classical analyses with bivariate plots were necessary to interpret the data (Fig. 3B–E). These observations confirm that upon treatment by SPADE analyses, homogeneous EV subpopulations were identified, and further highlight the complexity of analyzing high-dimensional flow cytometry data without appropriate computerized tools.

### Analysis of platelet response to thrombin stimulation using SPADE

**Figure 4:**
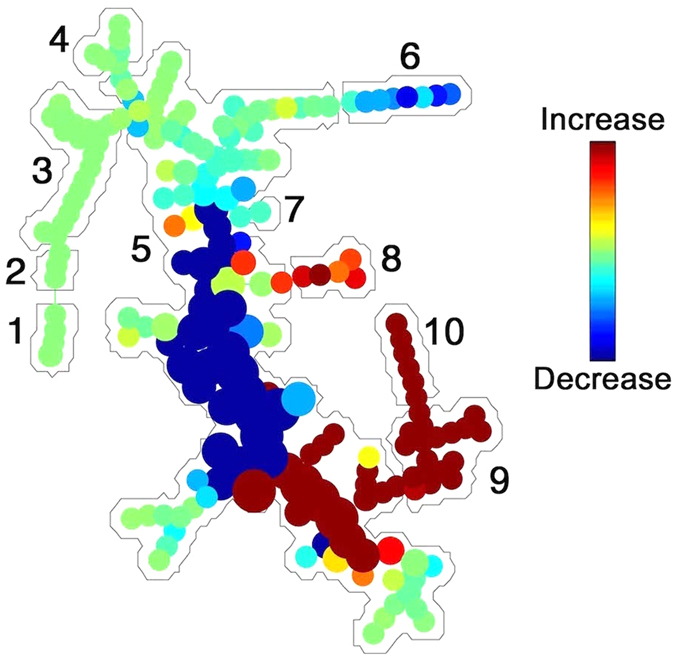
SPADE tree to assess EVs in cellular perturbations. After stimulation of platelets with thrombin, cell frequency decrease (blue) in platelets (1) and increase (red) in platelet EVs (9*–*10). Red blood cell markers (CD235a) (2,3), other cells (1), and noise/background (5) were mostly unchanged. Platelet CD41a^+^ EVs (9,10) and extracellular mitochondria lacking CD41a^+^ (*h*, naked mitochondria) were induced (red). Data are representative of 3 independent experiments.

EV production is evidence of cellular activation or apoptosis. Thus, the SPADE analysis as above was used to appreciate the platelet response to thrombin stimulation (Fig. 4). Both resting and activated platelets were portrayed in the constructed tree. The fold-change in subpopulation frequencies varied upon activation (*i.e*. red, increase; blue, decrease), indicating that platelet EV subpopulations (9*–*10), and extracellular mitochondria (8), were produced, while platelets (6) lost their dominance. Background/debris (5) varied following the stimulation, pointing to the generation of debris following platelet activation or the presence of unidentified EVs using this set of markers. These observations confirm the ability of SPADE to distinguish cell and EV populations, and to appreciate cellular plasticity and EV biogenesis in response to stimuli.

### Generating overlapping panels in plasma EV analyses

**Figure 5:**
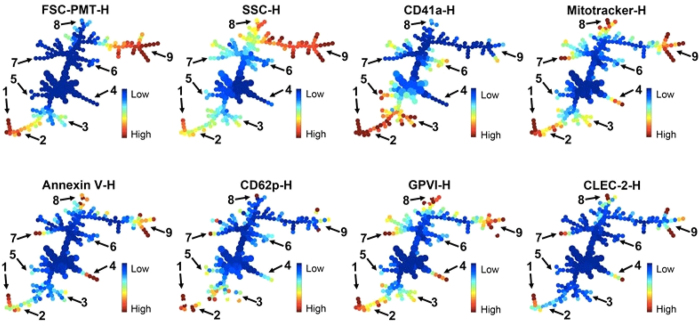
SPADE overlapping panels for EV diversity in plasma. EVs present in plasma were detected by hs-FCM, and FCS files were used to build a SPADE tree. Each tree is colored according to the indicated marker intensity. Mean fluorescence intensity ranging from low (blue) to high (red) for size (FSC-PMT-H), granularity (SSC-H), fluorescent-conjugated antibodies (CD41a^−^H, CD62P-H, GPVI-H, CLEC-2-H), MitoTracker and annexin V. Nine subpopulations were highlighted by manual identification based on the expression of the different markers, starting from the bottom of the tree: (1) Platelet EVs, positive for every marker (high intensity shown in red); (2) Platelet EVs positive for every marker except annexin V and CLEC-2 (low intensity shown in blue); (3) platelet EVs (CD41a high) with weak expression (shown in yellow) of MitoTracker and GPVI, and negative for annexin V, CD62P and CLEC-2; (4) EVs negative for CD41a, but positive for MitoTracker, annexin V and GPVI; (5) EVs presenting high expression of CD41a with low light scattering in FSC and SSC; (6) EVs with a weak expression of CD41a, negative for all other markers; (7) EVs negative for CD41a, and positive for all other markers, including activation markers (annexin V and CD62P); (8) EVs positive for every marker and presenting higher inner complexity (SSC-H) and (9) EVs negative for CD41a and presenting higher intensity in FSC-PMT. Data are representative of 3 independent experiments.

SPADE also permits the integration of multiple staining using overlapping marker panels (cocktail 5a-d in Table S1). For these experiments, we evaluated endogenous EVs present in healthy human platelet-free plasma samples and generated a new tree. The overlapping markers CD41a, MitoTracker, FSC-PMT and SSC, were present in every condition, and we also included annexin V, CD62P, GPVI and CLEC-2 as interchangeable markers within the tree (Fig. 5)^16^,^20^,^21^. FCS files were exported and analyzed as above. The SPADE parameters were the same except for the target densities that were fixed to 10,000 cells/EVs. Platelet-derived EVs (high CD41a expression) were mostly located at the bottom of the tree (the lower branches 1–3), some of them expressing GPVI and CLEC-2 (branches 1,2). Of note was that the upper part of the tree (CD41a^−^) also revealed high expression of GPVI and CLEC-2 on EVs (branches 7–9). Thus, the SPADE algorithm provides analyses of high-dimensional data that is scalable with an increasing number of markers useful for EV analysis. Furthermore, these data demonstrate that SPADE can identify subpopulations, like the presence of three subpopulations of platelet-derived EVs that could have been overlooked with classical dot plot analyses.

### SPADE for the appreciation of EVs as biomarker in disease

**Figure 6:**
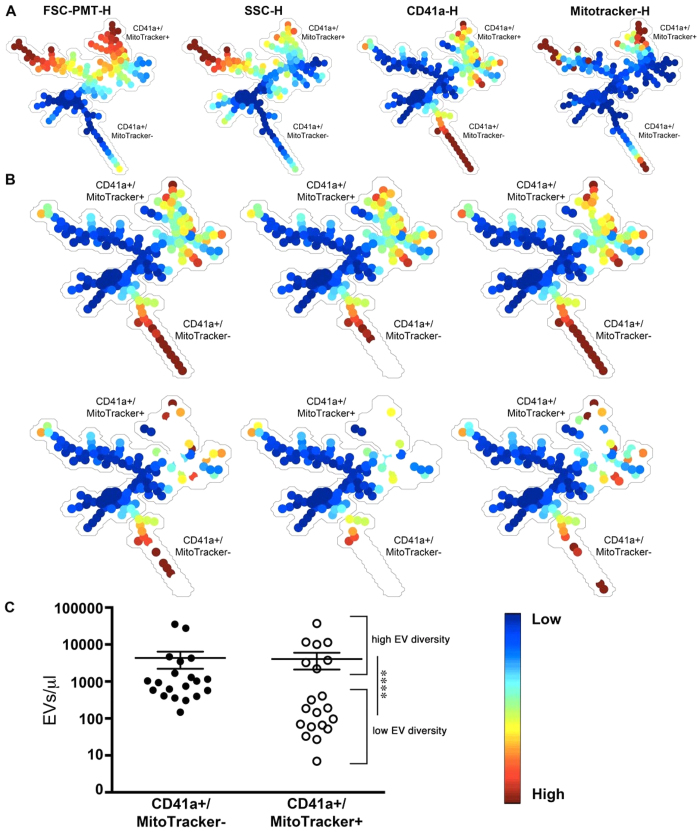
SPADE and EV-based biomarkers in disease. (**A**) Platelet-derived EVs present in synovial fluid (SF) of rheumatoid arthritis (RA) patients were detected by hs-FCM and portrayed in a SPADE tree (n = 20). Each tree is colored according to the indicated marker. Mean fluorescence intensity ranging from low (blue) to high (red) for size (FSC-PMT-H), granularity (SSC-H), fluorescent-conjugated antibodies CD41a-H and MitoTracker. (**B**) Representative tree of 6 RA synovial fluids with high (upper trees) or low (lower trees) EV diversity. (**C**) Platelet EVs containing mitochondria (CD41a^+^ MitoTracker^+^) and those not containing mitochondria (CD41a^+^ MitoTracker^−^) were detected among the 20 RA patients tested. ****P < 0.0001 between high and low EV diversity, Mann-Whitney test.

Different cellular lineages contribute to EV accumulation in the synovial fluid (SF) of RA patients. Platelet-derived EVs have been identified in RA SF^5^,^13^,^25^,^51^,^52^, and present with heterogeneous dimensions and mitochondrial content (Fig. 6A). We quantitatively identified EVs in the SF of 20 RA patients on the unique basis of 4 markers (*i.e*. CD41a, MitoTracker, FSC-PMT and SSC) to generate a new SPADE tree (Fig. 6A). SPADE parameters were the same as in the first tree (Figs 3 and 4) with the exception that 150 clusters were used instead of 200 given the reduced number of parameters measured. Using the autosuggestion tool to objectively identify EV subpopulations, two major subpopulations were revealed: *i.e*. CD41^+^, MitoTracker− and CD41^+^, MitoTracker^+^ EVs. SPADE tree highlighted the great variability between RA patients, as some EV subtypes appeared to be completely absent (empty nodes in white) in some patients (Fig. 6B,C). These differences in EV expression patterns did not seem to correlate with rheumatoid factor (RF) and anti-citrullinated protein auto-antibody (anti-CCP) levels (Table S2). These data suggest that EV pattern recognition using the objective analysis tool SPADE can highlight differences in EV content in disease, providing a tool for the determination of potent biomarkers in disease.

## Discussion

The emergence of EVs as important players in intercellular communication has opened the way to intensive research on this topic. EV levels are modulated in certain pathologies, and studies have established the vast diversity of EVs produced by cells, suggesting that EVs might be used as biomarkers^2^,^7^. Groups of scientists have established the most appropriate pre-analytical conditions for the study of EVs and for the design of modern methodologies for their fine characterization^3^,^9^,^10^,^11^,^13^,^14^,^17^,^18^,^26^,^27^,^29^,^30^,^48^,^49^,^50^. Furthermore, efforts have been made in recent years to institute a coherent nomenclature for EVs^44^. Not with standing these major improvements, it remains an obvious challenge to objectively interpret the large quantity of high-dimensional data in EV analyses. For instance, although fluorescence, rather than light scatter, used as trigger greatly improves EV detection, the distinction of EVs from background in FCM is still an obstacle in complex fluids^12^,^13^,^17^. Furthermore, platelets are abundant in blood and represent an important source of EVs; however, they are frequently misinterpreted as EVs given their relatively small dimensions^11^. The absence of such analytical tools for the comprehension of EV functions prompted our study.

SPADE offers the advantage of using an encompassing panel of markers to cluster the data, which allows the identification of rare cell types and facilitates new, unanticipated, biological discoveries^37^,^38^. Our study shows that SPADE is a versatile computerized tool to objectively handle hundreds of thousands of hs-FCM EV data and to reveal unpredicted EV subtypes. Most importantly, SPADE can be utilized for the analysis of EV data obtained with any flow cytometer, assuming that EVs are detected correctly. Whereas there exist other algorithms (other than SPADE) available for the interpretation of FCM data^53^, SPADE is among the most appropriate to reveal rare subpopulations of events by flow cytometry^37^,^38^,^43^. Investigators, however, need to compare trees from one condition to another to identify changes in nodes between conditions, which can be challenging if FCM-based biomarkers are examined in multiple patients, for example. Future improvements to these applications by engineers in the field might include high throughput tree comparison.

SPADE permitted the establishment of a tree that portrays EVs from platelets and RBC, the two main sources of EVs reported in blood. Of interest is that SPADE successfully recognized platelet activation based on platelet-derived EV production, suggesting that it might be used as a tool to assess EV-based cellular perturbations. Unanticipated subpopulations of EVs present in plasma were revealed by a second SPADE analysis with overlapping markers. Prior studies revealed that the majority of the platelet-derived EVs in blood in fact originate from MKs, the cells from which platelets are produced^16^,^20^. The immunoreceptor-based activation motif (ITAM) receptors GPVI and CLEC-2 were reported absent on the surface of platelet-derived EVs, but were found on the surface of (MK)-derived EVs, which also express CD41a^16^,^20^,^21^. Whereas SPADE might have revealed MK-derived EVs in plasma (CD41^+^ GPVI^+^ CLEC-2^+^ EVs), it unexpectedly shed light on CD41^−^-EVs expressing GPVI and CLEC-2. Although the functions of these EVs remain to be established, we hypothesize that these EVs might also originate from MKs. As for any data interpretation implicating flow cytometry, complementary approaches, such as electron microscopy, functional assays and biochemistry, should be utilized to confirm the actual presence of novel populations of EVs and to verify their biological significance.

The analysis of EVs contained in bio-specimens from disease patients permits the identification of biomarkers. With a rather simple analysis that included assessment of CD41a and mitochondrial content in EVs from 20 patients, a third SPADE analysis highlighted the heterogeneity that prevails in RA. The possibility to extract the number of events in each node for all patients, similarly to the exportation of statistics in traditional gating, allows a relatively easy comparison between patients. RA patients with higher EV diversity were usually those with higher platelet EV concentrations, pointing to a more important platelet contribution to the pathology in those patients. As it is suggested that platelet EVs invade the synovial space due to enhanced joint vascular permeability^54^, it might also provide information on the integrity of the vasculature in these patients. RA patients have enhanced risks of death due to cardiovascular disorders. Future studies might determine if increased levels of EV subtypes are an indication of certain comorbidities (other than those that were presented in Table S2) or impact response to different treatment in RA. Furthermore, we suggest that the combination of these approaches might be used for a qualitative and a quantitative stratification of patients suffering from heterogeneous diseases, such as RA and potentially other rheumatic diseases.

In this study, SPADE was applied to the analysis of high-dimensional data based on EV detection. We suggest that this approach may be utilized for the assessment of more numerous EV markers by FCM or mass cytometry. An in-depth understanding of EV subtypes modelled as high-dimensional point clouds will accelerate the implementation of EV subtypes as biomarkers and will facilitate the understanding of their role(s) in different contexts such as coagulation, inflammation, cancer, and immunity.

## Additional Information

**How to cite this article**: Marcoux, G. *et al*. Revealing the diversity of extracellular vesicles using high-dimensional flow cytometry analyses. *Sci. Rep*. **6**, 35928; doi: 10.1038/srep35928 (2016).

**Publisher’s note:** Springer Nature remains neutral with regard to jurisdictional claims in published maps and institutional affiliations.

## Supplementary Material

Supplementary Information
