# Supplementary material for: Revealing the diversity of extracellular vesicles using high-dimensional flow cytometry analyses
Source: Sci Rep. 2016 Oct 27;6:35928. doi: 10.1038/srep35928 (PMC5081512; doi:10.1038/srep35928)
Supplement: Supplementary Information [file srep35928-s1.doc]

**Supplementary Info**

**Revealing the diversity of extracellular vesicles using high-dimensional flow cytometry analyses**

Geneviève Marcoux, Anne-Claire Duchez, Nathalie Cloutier, Patrick Provost, Peter A. Nigrovic and Eric Boilard

**
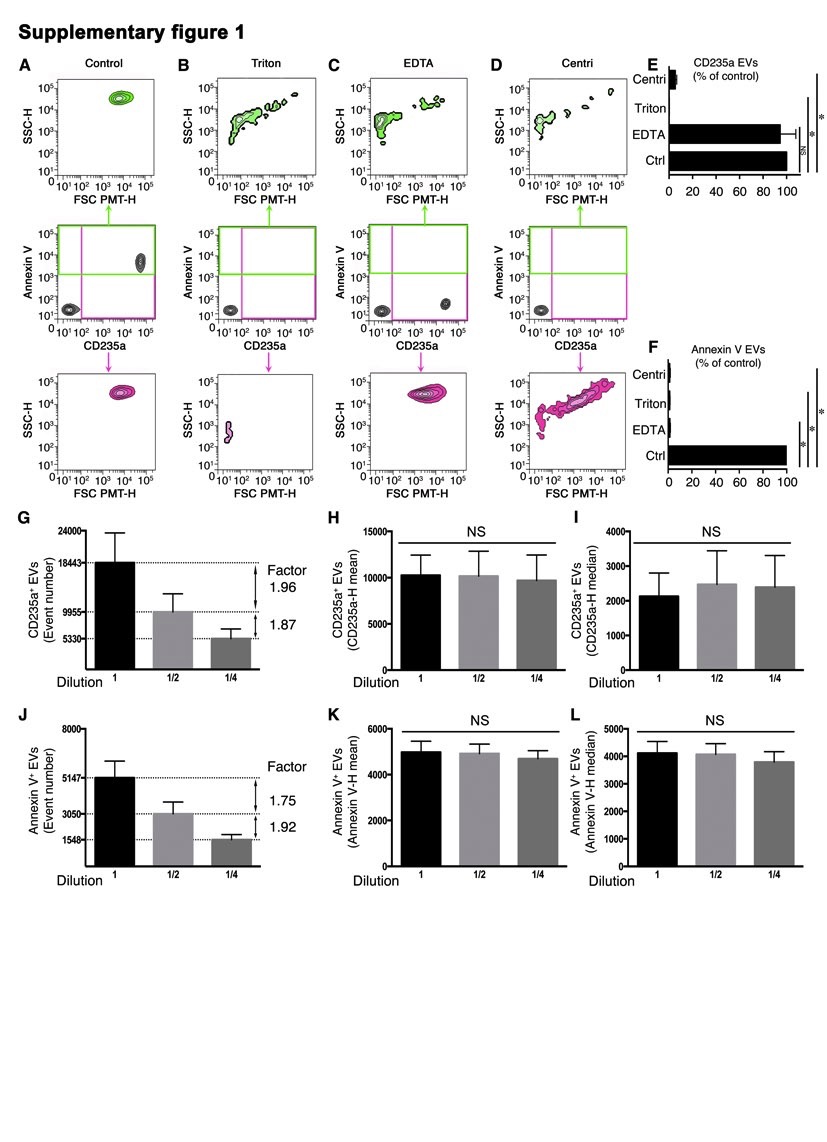
**

**Supplementary Fig. 1: Detection of red blood cell subpopulations by hs-FCM.**

(A) Representative SSC-H (granularity) and FSC-PMT-H (relative size) dot plots of RBC EVs detected using annexin V and fluorochrome-conjugated antibodies directed against CD235a in the absence of treatment (control). (b–d) FSC-PMT/SSC portrayals of RBC EVs detected with fluorochrome-conjugated annexin V and fluorochrome-conjugated antibodies directed against CD235a and treated with 0.05% Triton X-100, (B) 50 μM EDTA, (C) or after clearance of EVs using ultracentrifugation (centri)(D). Total annexin V+ events are presented in the green gate (middle panel) and their relative size and granularity is displayed in the upper panel. Total CD235a+ events are presented in the blue gate (middle panel) and their relative size and granularity is displayed in the lower panel. Data are representative of 3 independent experiments. (E–F) Sensitivity of CD235a (E) and annexin V (F) EVs to clearance by ultracentrifugation (centri), Triton and EDTA, presented as % of untreated (Ctrl). (G–L) CD235a+ and annexin V+ EVs were serially diluted twice (2-fold dilution) and quantitatively analyzed by hs-FCM using counting microspheres. Their concentration (G and J), the mean of fluorescence (H and K) and the median of fluorescence (I and L), are presented. Data are presented as the mean ± SEM of 3 independent experiments, *P < 0.05 compared with the control (Ctrl); NS : Non significant ; Wilcoxon test.

**
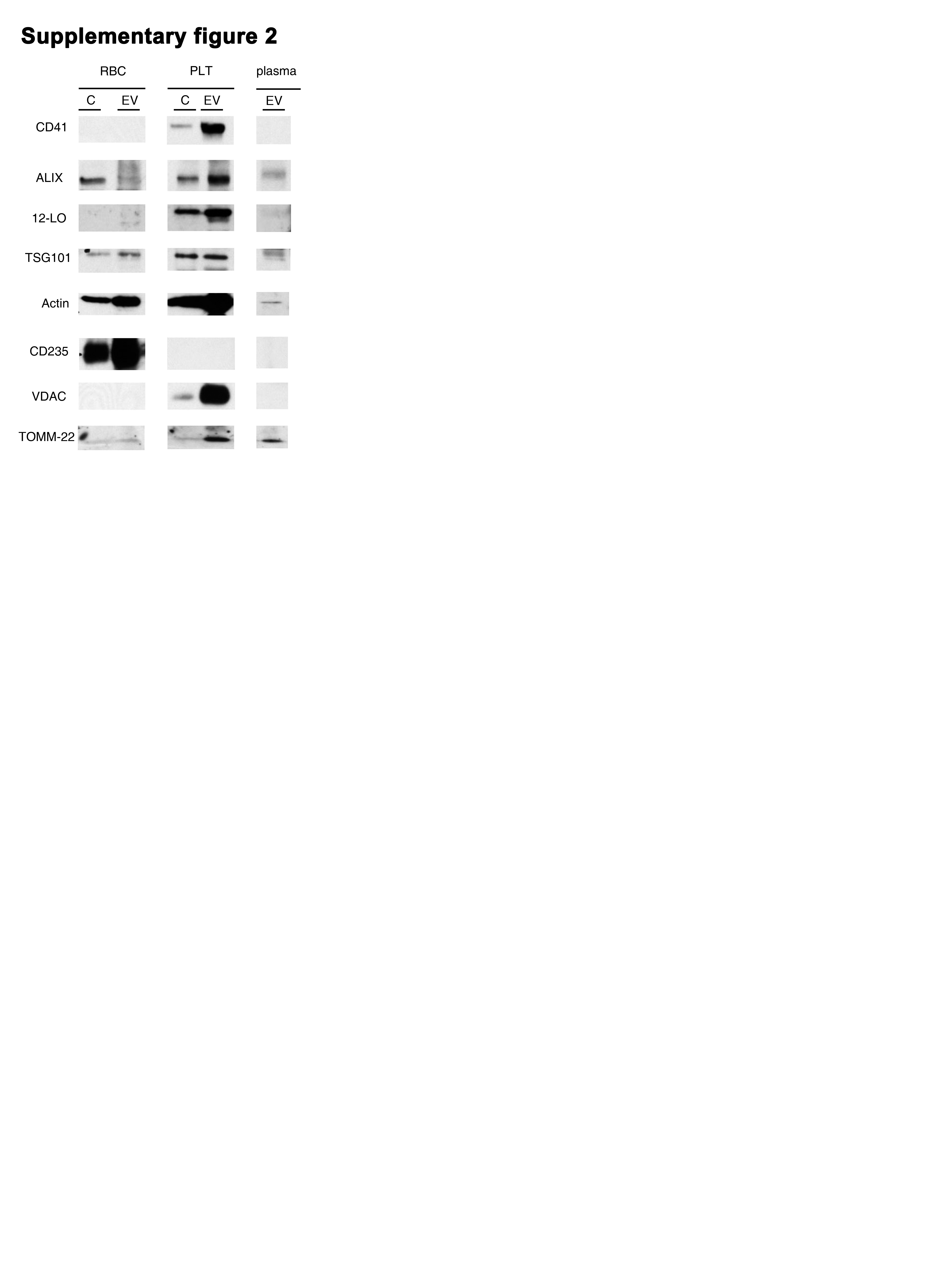
**

**Supplementary Fig. 2: Protein content in cells and EVs from plasma, platelets and RBC.**

Immunoblot of CD41a, ALIX, 12-LO, TSG101, actin, CD235a, VDAC and TOMM-22 in cells (C) and EVs (EV) from platelets (PLTs), red blood cells (RBC) and plasma. Data are representative of three independent experiments. Cells (C), EVs (EV).

**
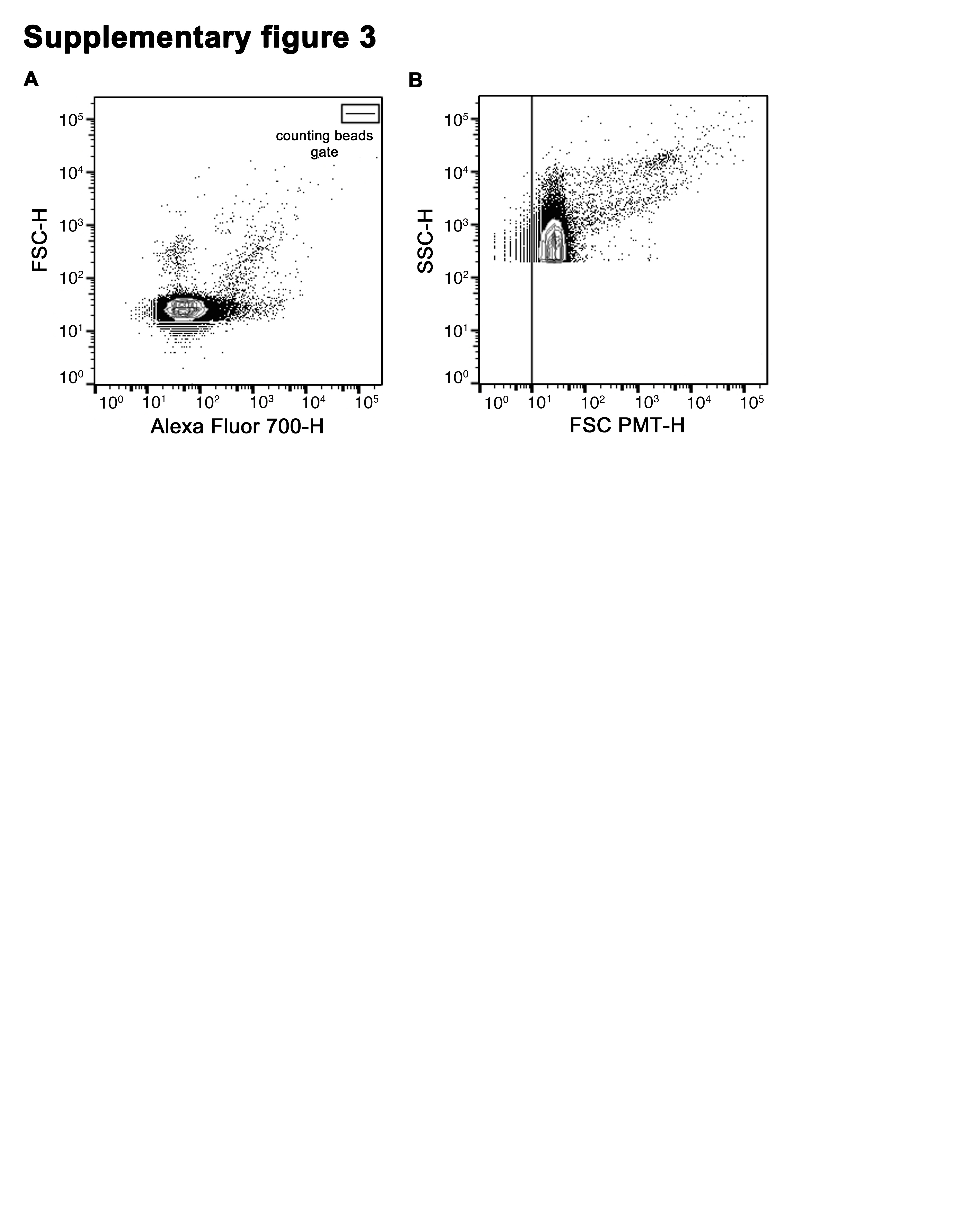
**

**Supplementary Fig. 3: Initial gating before performing SPADE analysis.**

(A) After the acquisition of fluorescent signals, an initial gating was performed on all data to exclude counting beads from files. (B) After bead exclusion, a gate was drawn to

(B) Include events over 100 nm in diameter (based on silica beads from Fig. 1B).

**Supplementary Table 1 : Staining panels for the different SPADE tree***

| **Figure** | **Antibody/label** | **Suppliers** | **Concentration** | **Labelling buffer** |
| --- | --- | --- | --- | --- |
| **3-4** | V450-conjugated anti-human CD41a | BD Bioscience, clone HIP8 | 1 µL | Annexin V buffer (BD Pharmingen) pre-filtered through a 0.22-µm-pore-size membrane (Fisher Scientific, ON, Canada). |
|  | PE-Cy7-conjugated anti-human CD235a | BD Pharmingen, clone HIR2 | 3 µL |  |
|  | FITC-conjugated annexin V | BD Pharmingen | 3 µL |  |
|  | MitoTracker Deep Red (APC)** | Life Technologies | 100 nM |  |
| **5a** | V450-conjugated anti-human CD41a | BD Bioscience, clone HIP8 | 1 µL | Annexin V buffer pre-filtered through a 0.22-µm-pore-size membrane |
|  | MitoTracker Green (FITC) | Life Technologies | 100 nM |  |
|  | APC-conjugated anti-human C-type lectin receptor 2 (CLEC-2) | R&D System, clone 219133 | 10µL |  |
| **5b** | V450-conjugated anti-human CD41a | BD Bioscience, clone HIP8 | 1 µL | Annexin V buffer pre-filtered through a 0.22-µm-pore-size membrane |
|  | MitoTracker Green (FITC) | Life Technologies | 100 nM |  |
|  | PE mouse anti-human platelet glycoprotein VI (GPVI) | BD Pharmingen, clone HY101 | 1 µL |  |
| **5c** | V450-conjugated anti-human CD41a | BD Bioscience, clone HIP8 | 1 µL | Annexin V buffer pre-filtered through a 0.22-µm-pore-size membrane |
|  | MitoTracker Green (FITC) | Life Technologies | 100 nM |  |
|  | APC-conjugated annexin V | BD Pharmingen | 3 µL |  |
| **5d** | V450-conjugated anti-human CD41a | BD Bioscience, clone HIP8 | 1 µL | Annexin V buffer pre-filtered through a 0.22-µm-pore-size membrane |
|  | MitoTracker Green (FITC) | Life Technologies | 100 nM |  |
|  | APC-conjugated anti-human CD62P | BD Pharmingen, clone AK-4 | 3 µL |  |
| **6** | PE-conjugated anti-human CD41a | BD Bioscience, clone HIP8 | 1 µL | PBS pre-filtered through a 0.22-µm-pore-size membrane |
|  | MitoTracker Deep Red (APC) | Life Technologies | 100 nM |  |

*All staining were performed at 37°C for 30 minutes.

** MitoTracker was omitted for the RBC derived samples.

| **Gender** | **Age (years)** | **RF** | **Anti-CCP** | **ESR (mm)** | **CRP level (mg/L)** | **CD41+MitoTracker+ EVs** | **CD41+ EVs** |
| --- | --- | --- | --- | --- | --- | --- | --- |
| N/A | N/A | N/A | N/A | N/A | N/A | 3751 | 358 |
| F | 77 | 2720 | N/A | 90 | 17.4 | 11652 | 1671 |
| F | 75 | 67 | N/A | 22 | 20.5 | 2214 | 4296 |
| F | 78 | 2720 | N/A | 71 | 9.0 | 11369 | 3460 |
| F | 88 | 2000 | N/A | 85 | 39.9 | 37171 | 4664 |
| F | 71 | 96 | 10 | 14 | 5.6 | 3249 | 27513 |
| F | 71 | 96 | 10 | 14 | 5.6 | 10040 | 35269 |
| F | N/A | N/A | N/A | N/A | N/A | 27 | 398 |
| F | 56 | 25 | N/A | 56 | 35.1 | 318 | 1031 |
| M | 62 | <10 | 1 | 29 | 5.0 | 7 | 147 |
| F | 34 | 205 | N/A | 74 | 4.3 | 191 | 573 |
| M | 34 | <10 | 1 | 26 | 19.3 | 52 | 306 |
| M | 63 | 94 | 1 | 12 | 6.6 | 144 | 582 |
| F | 76 | 392 | N/A | 24 | 9.4 | 186 | 933 |
| F | 65 | N/A | N/A | 3 | 94.7 | 97 | 623 |
| F | 65 | N/A | N/A | 3 | 94.7 | 66 | 407 |
| F | 63 | 48 | >100 | 25 | 11.6 | 70 | 1288 |
| F | 60 | 324 | 100 | 42 | 11118.6 | 33 | 747 |
| F | 59 | 173 | >100 | 40 | N/A | 403 | 1152 |
| M | 59 | 289 | N/A | 10 | 4.1 | 59 | 1022 |

**Supplementary Table 2. Demographic and clinical characteristics of RA patients.**

Synovial fluid is from affected joint (knee). Different parameters were measure as gender, age, Rheumatoid Factor (RF), anti-cyclic citrullinated peptide (CCP), erythrocyte sedimentation rate (ESR), C-reactive protein (CRP), CD41+MitoTracker+ EVs and CD41+ EVs.
